# Emotional modulation of temporal density in natural grasping: a vision-based kinematic study

**DOI:** 10.3389/fpsyg.2026.1823532

**Published:** 2026-06-17

**Authors:** Hakki Egemen Gülpinar, Anastasiia Skryzhadlovska, Robert Haschke, Olga Abramov, Amir Jahanian Najafabadi

**Affiliations:** 1 Department of Technology, Bielefeld University, Bielefeld, Germany; 2 Department of Cognitive Neuroscience, Bielefeld University, Bielefeld, Germany

**Keywords:** affective motor responses, emotion recognition, grasping behavior, hand kinematics, human-object interaction, object interaction

## Abstract

**Introduction:**

While emotions affect perception, their influence on physical interaction remains underexplored. This study investigated how the emotional valence of everyday objects manifests in hand kinematics during natural grasping.

**Methods:**

Hand movements were recorded from 40 participants using an overhead smartphone camera (1080p; 30 fps), combined with MediaPipe and OpenCV. Participants were randomly assigned to either a Visual Modality (*n* = 20) or a Non-Visual Modality (*n* = 20) and were instructed to grasp five objects designed to evoke distinct emotional responses (fear, disgust, neutrality, and happiness). Kinematic measures, including grasping frame rate (frames/s) and hand velocity (px/frame), were extracted alongside self-reported emotional ratings.

**Results:**

Results revealed that motor behavior is not merely functional but emotionally reactive: threat-based objects (e.g., a spider) elicited significantly higher grasping frame rates, indicating more sustained interaction with these stimuli, potentially reflecting heightened monitoring or cautious engagement. A linear mixed-effects model identified a significant Modality × Object interaction, indicating that visual access influenced object-related effects selectively rather than uniformly. Notably, these affect-related changes were captured in grasping frame rate (a temporal-density measure) rather than in hand velocity.

**Discussion:**

The emotional properties of objects systematically modulate motor behavior during grasping. These findings help bridge the gap between affect and physical action. They also show that consumer-grade smartphone technology can detect subtle variations in natural hand interaction, opening the door for affect-aware interfaces and scalable studies of motor-affective behavior in naturalistic environments.

## 1 Introduction

The human hand is not merely a tool for physical interaction but also a subtle medium of emotional expression. Prior research demonstrated that hand kinematics respond not only to object features such as weight, texture, and temperature, but also to the emotional context of interaction. Lederman and Klatzky (1987) reported that exploratory hand movements are systematically linked to the extraction of object properties during haptic recognition. More recent work revealed that emotional valence modulates movement dynamics. For example, Esteves et al. (2016) reported systematic changes in peak velocity and movement duration when participants interacted with emotionally charged stimuli. Similarly, Nogueira-Campos et al. (2016) showed that observing grasping actions directed at emotion-laden objects modulates motor system responses, supporting the concept of embodied emotion.

Understanding the emotional component of grasping is crucial in domains such as social robotics, affective computing, rehabilitation, and human-computer interaction, where affect-sensitive physical interaction plays a central role (Culbertson et al., 2018). The interplay between emotion and action is extensively explored through facial expressions, vocal cues, and body posture, with studies demonstrating that emotions shape motor patterns and embodied expressions (Esteves et al., 2016; Nogueira-Campos et al., 2016; Culbertson et al., 2018; Winkielman et al., 2015). Research in embodied cognition further emphasizes that emotions influence not only perception but also the planning and execution of actions, highlighting the tight coupling between affective states and motor behavior (Winkielman et al., 2015). Recent task-relevance accounts further suggest that emotional stimuli influence action most reliably when they are directly relevant to the current goal (Montalti and Mirabella, 2023; Mirabella and Montalti, 2025). This perspective is especially relevant here because the emotional object was also the target of the grasping action, rather than a task-irrelevant background cue.

While these modalities receive significant attention in emotion research, the nuanced expressiveness embedded in hand movements, especially during functional acts like grasping, remains underexplored. In this work, we address this gap by investigating how natural emotional responses to objects shape hand kinematics during grasping. We employed a non-intrusive method using a regular smartphone camera in combination with MediaPipe (Lugaresi et al., 2019) and OpenCV (Kaehler and Bradski, 2017) to track and analyze hand motion, enabling data collection in a naturalistic and accessible manner. Participants interacted with five objects designed to evoke distinct emotional responses (e.g., neutrality, happiness, disgust, and fear), and self-reported feedback was collected after each trial to link movement patterns with subjective emotional states. The contribution of the study is threefold: it shifts the unit of analysis from the initial reach phase to continued hand-object interaction, uses real physical objects rather than abstract or screen-based stimuli, and evaluates whether a consumer-grade smartphone with a landmark-based computer-vision pipeline can detect affect-related differences in natural grasping behavior.

The present study was guided by two closely related questions. **First**, we examined whether objects that evoke different emotional responses elicit systematically distinct hand kinematics during natural grasping. **Second**, we examined whether these differences are more apparent in movement speed or in grasping frame rate.

These questions were tested under two experimental modalities: one where participants could not see the objects (isolating tactile perception) and one where they could (including visual-emotional anticipation). This design allowed us to assess both the conscious and subconscious influence of emotion on hand movement.

## 2 Related work

This study builds directly on the foundational work of Lederman and Klatzky (1987), who demonstrated that hand movements during object exploration are highly structured and tied to specific perceptual goals. They introduced the concept of exploratory procedures, which are stereotyped movement patterns such as lateral motion, pressure, static contact, contour following, and enclosure that are intentionally used to extract object properties like texture, hardness, temperature, shape, and weight. Through controlled experiments, they showed that participants consistently selected the most efficient and informative hand movements based on the perceptual dimension being explored.

This framework continues to shape our understanding of haptic exploration by emphasizing that hand movements are not random or purely biomechanical, but intentional and cognitively driven. Recent studies, such as Esteves et al. (2016), extended this idea into the domain of affective interaction by demonstrating that emotional valence systematically influences motor behavior. Their findings revealed that emotional valence modulates reach-to-grasp kinematics, with pleasant stimuli facilitating movement execution through an increased time-to-peak-velocity ratio, indicating that affect shapes the temporal dynamics of action planning. Whereas, Esteves et al. (2016) focused primarily on classical reach-to-grasp kinematics during the approach phase of movement, the present study examines continued hand-object interaction with real three-dimensional objects and includes a Non-Visual Modality. The current design is therefore complementary to prior reach-to-grasp work rather than a direct replication.

Building on these insights, the current research investigated whether emotion influences not only the timing but also the overall pattern and quality of grasping movements. Unlike prior studies that focused on isolated motor parameters in constrained settings, we employed a naturalistic, vision-based approach using MediaPipe (Lugaresi et al., 2019) and OpenCV (Kaehler and Bradski, 2017). This methodological choice is consistent with the state-of-the-art in the field, as computer vision-based hand gesture recognition has become a cornerstone for affective computing and human-robot interaction (Qi et al., 2024). This approach enables the capture of rich hand kinematic data during spontaneous interactions with emotionally evocative objects. Complementing our pipeline choice, a registerable, MediaPipe-based real-time gesture system shows that landmark-only inputs combined with metric-learning approaches can flexibly cluster and register novel gestures, supporting scalable camera-only hand analysis (Meng et al., 2024). Converging with our focus on hand/pose landmarks, recent work demonstrated that body- and hand-pose features alone can support multi-class emotion recognition on controlled datasets (e.g., GEMEP), underscoring the informativeness of kinematic cues (Martinez-Martin and Fernández-Caballero, 2025).

Moreover, our study aligns with recent discussions in affective computing and embodied cognition. Nogueira-Campos et al. (2016) reported that observing grasping actions directed at emotion-laden objects activates distinct motor responses in the observer, supporting the existence of emotion-specific motor signatures. Similarly, Winkielman et al. (2015) and Culbertson et al. (2018) emphasized that emotion and motor control are deeply intertwined, with implications for both artificial systems and human-computer interaction. Within HRI, comprehensive reviews synthesize camera-based hand-gesture recognition methods and highlight design trade-offs relevant to our vision-only kinematic analysis (Qi et al., 2024). Despite this growing body of research, relatively few studies have explicitly investigated how emotional responses influence natural grasping behavior in real-world scenarios. By integrating subjective emotional reports with computer vision-based motion analysis, we aim to bridge this gap and contribute to both haptic cognition and affect-sensitive motor research.

### 2.1 Current study

In this study, we investigated how natural emotional responses evoked by various objects are reflected in hand kinematics during grasping. We examined this under two experimental modalities: one where participants could visually perceive the objects while grasping, and another where visual information was withheld. Building upon established frameworks linking affective appraisal to motor execution (Lederman and Klatzky, 1987; Esteves et al., 2016), this study was guided by two primary hypotheses. First, we hypothesized that the emotional valence of an object would alter the physical interaction, such that objects evoking distinct emotional responses would manifest in significantly different hand movement patterns, specifically within the dimensions of movement velocity and grasping frame rate. Second, we predicted that emotionally salient objects would elicit more dynamic grasping behavior than neutral or comfort-associated objects. Together, these design choices allowed us to examine affect-related grasping behavior during sustained contact with real objects, rather than only during the initial transport phase of a reach.

## 3 Methods and procedure

### 3.1 Participants

A sample of forty healthy young adults (*N* = 40; age range 18–30 years) was recruited from the student population at Bielefeld University. To ensure a standardized motor profile, all participants were right-handed and exhibited normative motor function, with no self-reported history of musculoskeletal, neurological, or psychiatric disorders and relevant medications intake. Prior to the commencement of the experiment, participants provided written informed consent in accordance with the Declaration of Helsinki. To prevent behavioral bias, all individuals were naive to the specific study hypotheses and the emotional categories associated with the stimuli. The experimental protocol was reviewed and formally approved by the Ethics Committee of Bielefeld University (EUB-2025-217-S), ensuring compliance with institutional and international ethical standards for human research.

### 3.2 Experimental design

We employed a between-subjects design to evaluate the influence of sensory modality on affective motor control. Forty participants were equally distributed across two experimental groups. In the Visual Modality, participants interacted with objects placed openly on the workspace, allowing for full visual access and emotional anticipation prior to the motor act. Conversely, in the Non-Visual Modality, stimuli were positioned behind an opaque barrier with a recessed aperture. Participants inserted their hand through this opening to grasp the object, effectively isolating tactile perception from visual cues.

This configuration was specifically designed to preserve the element of surprise and mitigate habituation or familiarity effects. Each participant completed five trials, consisting of a single interaction with each of the five distinct stimuli. To ensure internal validity and minimize potential order effects or systematic biases, the sequence of object presentation was randomized for every subject. Participants remained naive to the specific nature of the objects and their intended emotional valence prior to the start of the experiment. This ensured that, particularly in the Non-Visual Modality, the initial motor planning was devoid of prior visual or conceptual knowledge, relying solely on emergent haptic feedback. After each trial, a brief emotional survey was administered to capture the subjective affective experience of the interaction.

### 3.3 Stimuli

**Figure 1:**
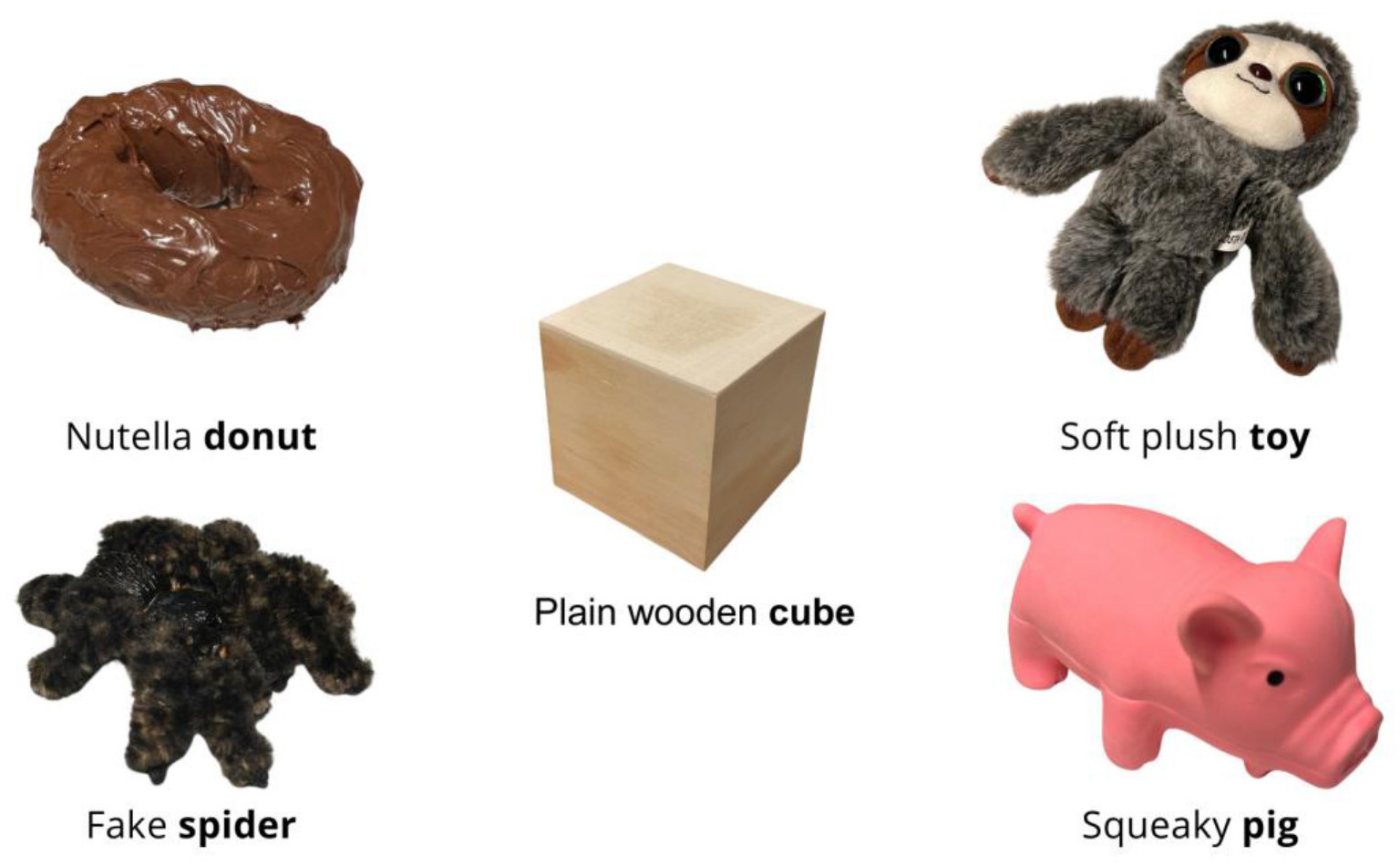
Stimuli used in the study.

Five objects were selected to elicit distinct emotional responses (Figure 1).

- Wooden box/cube (Neutral).
- Soft plush toy (Happiness/Comfort).
- Fake spider (Fear).
- Nutella donut (Disgust).
- Squeaky pig toy (Happiness/Surprise).

The objects were chosen to be physically recognizable and safely graspable while preserving relevant tactile properties. They were intended as naturalistic affective stimuli rather than perfectly standardized category markers. Briefly, the neutral wooden object was rigid, the plush toy was soft, the spider had a textured surface, the donut had a soft surface with a creamy topping, and the pig toy was soft and squeaky when squeezed.

### 3.4 Setup and recording

**Figure 2:**
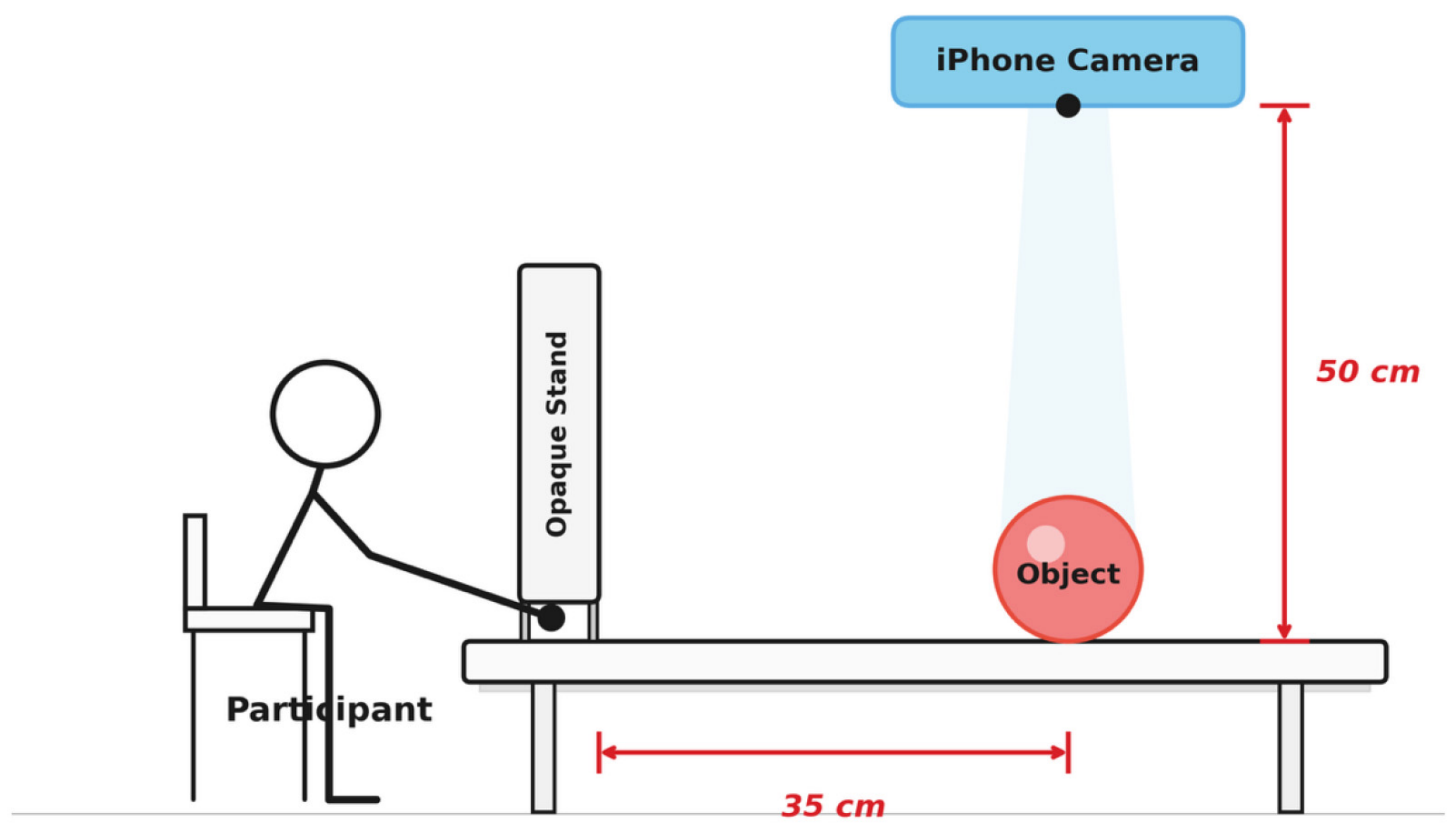
Schematic illustration of the experimental setup for the non-visual modality. The setup for the visual modality was identical, except that the opaque stand was removed. The camera height (approximately 50 cm), object distance (approximately 35 cm), and interaction region were kept constant across recordings.

The experimental environment was standardized to ensure spatial and temporal consistency across all trials. Stimuli were positioned at a distance of approximately 35 cm from the participant's starting position on a table surface aligned with fixed spatial markers. A fixed in-frame workspace region was marked with tape on the table surface and used as a consistent spatial anchor across participants. Hand kinematics were captured using an overhead vision-based system consisting of a high-definition smartphone camera (iPhone 14, 1,080p resolution) oriented perpendicular to the workspace. The device was secured on a tripod-based mount at a fixed altitude of approximately 50 cm, providing a comprehensive bird's-eye view of the interaction zone (Figure 2). The same phone, camera resolution, approximate camera height, object distance, and marked workspace region were maintained across recordings. To maintain strict temporal synchronization, source videos were acquired at 30 fps. To account for potential hardware-induced jitter and to ensure a unified temporal resolution across all participant datasets, recordings were processed at 30 fps. All subsequent landmark extraction via MediaPipe and OpenCV-based kinematic calculations were performed on these homogenized video streams, ensuring that the derived grasping frame rate and velocity metrics remained comparable across the entire cohort. Velocity was computed from frame-to-frame displacement of the tracked hand center in the video plane and reported in px/frame. Because all recordings used the same phone, resolution, 30 fps sampling rate, tripod-mounted camera height, object distance, and marked workspace region, this measure was used for within-study comparisons rather than converted to physical units such as cm/s.

### 3.5 Study datasets

Kinematic data were extracted from hand movements recorded by the camera. MediaPipe (Lugaresi et al., 2019) and OpenCV (Kaehler and Bradski, 2017) were used to analyze hand motion and compute key kinematic measures, including:

- Grasping frames (count): number of frames classified as grasp-positive within a trial. Grasp-positive frames were defined using a multi-metric threshold involving finger-to-thumb distance, hand openness, and finger curvature; the required number of close fingers depended on whether the frame met the holding or strong-grasping criteria. This definition encompasses both tight grips (strong grasping) and manipulative holding. For the primary dependent variable (grasping frame rate), both types are counted together as grasp-positive.
- Grasping frame rate (frames/s): grasp-positive frames divided by the hand detection time span in seconds (computed from the 30fps video stream).
- Hand velocity (px/frame): frame-to-frame displacement of the tracked hand center in the video plane; average and maximum velocity summaries were retained descriptively.
- Movement category distribution (%): for a finer-grained analysis, grasp-positive frames were further separated into grasping (tight grip) and holding (sustained contact), alongside other movements.

Importantly, this grasping measure reflects frame-level grasping activity over time and should not be interpreted as the number of discrete grasp episodes. In this paper, temporal density refers to the rate at which grasp-positive frames occur during the hand-detection interval, operationalized as grasping frame rate (frames/s).

**Figure 3:**
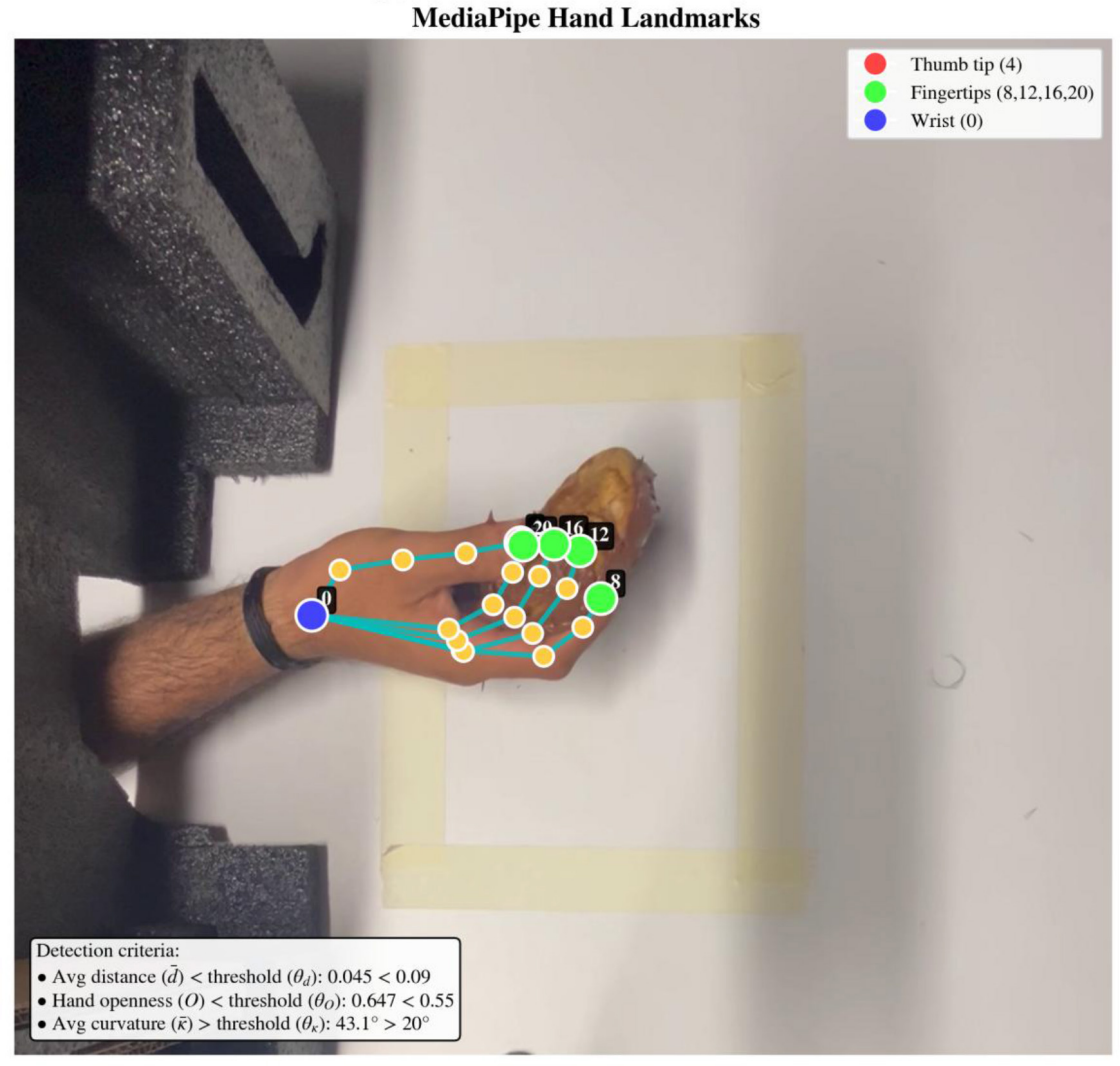
MediaPipe hand landmark detection on a sample grasping frame. Key landmarks are numbered: wrist (0), thumb tip (4), and fingertips (8, 12, 16, 20). The detection criteria box shows the computed metric values at this frame.

**Figure 4:**
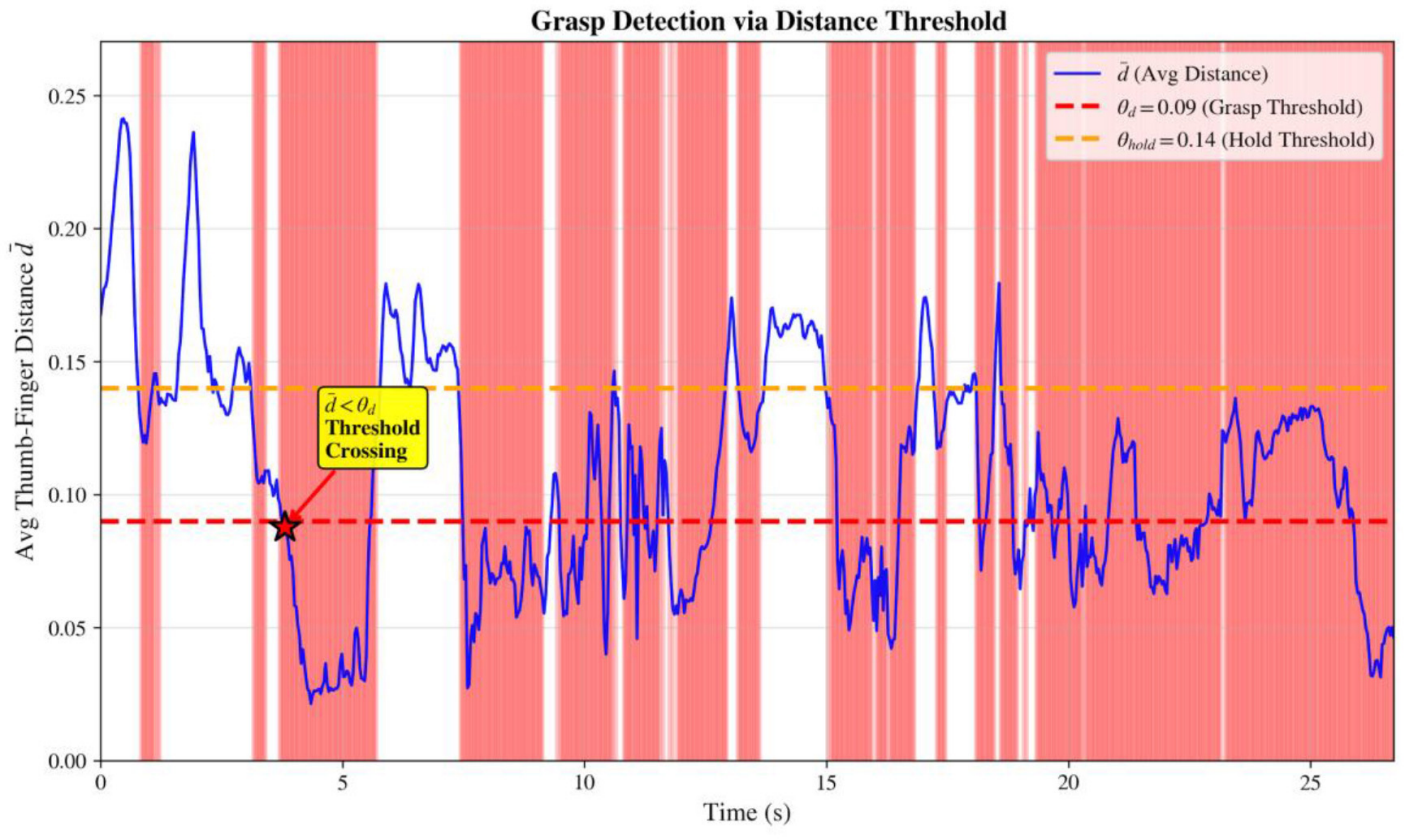
Time series of average thumb-to-finger distance during a sample trial (donut object). The **red dashed line** indicates the grasping threshold (_d_ = 0.09). **Red shaded** regions mark frames classified as grasping. The star indicates the first threshold crossing moment.

Figures 3, 4 illustrate the grasp detection methodology. Figure 3 shows a sample video frame with MediaPipe's 21-point hand landmark model overlaid, with key landmarks numbered. Figure 4 displays the corresponding time series of average thumb-to-fingertip distance, demonstrating how threshold crossings trigger grasp frame classification.

Subjective ratings were additionally recorded for each object to link emotional responses with kinematic features. Following each grasping trial, participants provided feedback through a survey capturing both qualitative and quantitative aspects of emotion. Participants first identified their primary emotion from: Neutral, Happiness, Surprise, Fear, Disgust, Frustration, Sadness, Confusion, Boredom, Nervousness, or Other (with optional description). For each reported emotion, participants then rated:

- Intensity (Low, Moderate, High).
- Comfort level during grasping (Very comfortable, Neutral, Uncomfortable).
- Familiarity with the object (Very familiar, Somewhat familiar, Not familiar at all).

In this paper, intensity refers to this ordinal post-trial self-report, whereas arousal refers to the broader theoretical affective construct; the two terms were therefore not treated as interchangeable. Positive and Negative Affect Schedule (PANAS; Watson et al., 1988) ratings were also summarized for each object as Positive Affect and Negative Affect scores, providing a dimensional complement to the categorical post-trial reports. Subjective ratings were used descriptively as manipulation-check information. The primary inferential models were based on object identity and modality rather than self-report labels, and trials were retained even when a participant's reported emotion differed from the intended object category.

### 3.6 Statistical analysis

To account for the hierarchical structure of the data, wherein repeated measurements (across five objects) were nested within individual subjects, we employed linear mixed-effects models (LMMs). This approach allowed us to model within-participant dependencies while capturing both fixed and random sources of variance. For the primary dependent variable, grasping frame rate (frames/s), we specified Modality (Visual vs. Non-Visual), Object (box, donut, pig, spider, toy), and their interaction as fixed effects. A random intercept for Participant-ID was included to control for individual differences in baseline motor activity:


$$\begin{array}{l} GraspingFrameRate\sim Modality\times Object+\left(1|ParticipantID\right) \end{array}$$

Model parameters were estimated using maximum-likelihood fits. The significance of the interaction term was evaluated via a likelihood-ratio test comparing the full interaction model against a reduced, no-interaction model. To ensure the stability of our findings, we performed a robustness check using an Ordinary Least Squares (OLS) model with participant-clustered standard errors. We additionally report mixed ANOVA results (Modality as a between-subjects factor, Object as a within-subjects factor) to allow comparison with prior literature that commonly uses this format. A Mann–Whitney *U*-test on participant-level means was included as a non-parametric robustness check, since it makes no distributional assumptions. In the event of a significant interaction, *post-hoc* modality contrasts within each object were conducted, with *p*-values adjusted using the Holm-Bonferroni correction to maintain family-wise error rates across the five stimuli. The identical modeling framework was applied to the analysis of hand velocity (px/frame) to maintain consistency across kinematic metrics.

To assess the strength of evidence for the reported effects relative to the modest sample size, we additionally conducted a Bayesian Model Comparison following the protocol of recent research (Jahanian Najafabadi et al., 2026). Two Bayesian Repeated Measures ANOVAs were performed in JASP (version 0.96), one for grasping frame rate and one for hand velocity, each with Modality (Visual/Non-Visual) as a between-subjects factor and Object (Box, Donut, Pig, Spider, Toy) as a within-subjects factor. We used default Jeffreys-Zellner-Siow (JZS) priors as implemented in JASP and compared all possible models (main effects and interactions) against the null model. Evidence is reported as the posterior model probability *P*(*M*|data), the change-from-prior factor BF_*M*_, and the inclusion Bayes Factor BF_*inclusion*_ for each effect. In cases where the null model was the best-fitting model, we additionally report BF_01_ (the inverse of BF_10_) to quantify evidence in favor of the null hypothesis. Bayes Factors were interpreted following (Jeffreys, 1961).

## 4 Results

### 4.1 Data overview (unified dataset)

We analyzed the unified dataset (see Section 3.1) obtained by combining Dataset 1 (ID01-ID20) and Dataset 2 (ID21-ID40). This resulted in 40 participants (20 Visual and 20 Non-Visual) and 200 object-level observations (five objects per participant). All videos were recorded at 1,080p and processed at 30 fps. Grasping frame rate (frames/s) was defined as the number of frames per second classified as grasping based on MediaPipe landmarks and a fingertip-distance threshold (see Methods).

### 4.2 Descriptive summary by object

Table 1 provides an overview of object-wise summary statistics. Across all participants, the spider elicited the highest mean grasping frame rate (*M* = 20.97 ± 7.17 frames/s), followed by the toy (*M* = 15.52 ± 5.80) and box (*M* = 12.79 ± 5.75). The donut (*M* = 11.48 ± 7.46) and pig (*M* = 11.43 ± 6.22) showed lower grasping frame rate on average. The most common self-reported emotions showed the following modal pattern: box (neutral), donut (disgust), spider (fear), and pig/toy (happiness). Across the 40 participants, the modal self-reported labels were Neutral for the box (25/40), Disgust for the donut (18/40), Fear for the spider (12/40), and Happiness for the toy (25/40), while the pig showed a more mixed positive profile dominated by Happiness (12/40) and Surprise (9/40). Positive and Negative Affect Schedule (PANAS) ratings showed a similar descriptive pattern: Negative/Positive Affect means were 1.32/2.14 for the box, 2.43/2.38 for the donut, 1.68/2.85 for the pig, 2.11/2.52 for the spider, and 1.35/2.63 for the toy. This ordering does not follow a simple valence gradient: the donut, despite evoking high-intensity disgust, showed low grasping activity, while the toy, associated with positive affect, ranked second highest. The spider's mean grasping frame rate was nearly double that of the donut and pig, suggesting that threat-related cues elevated sustained grasp-related contact.

**Table 1:** Enhanced summary statistics by object type (all participants).

Object	Avg grasping frames	Avg grasping frame rate (frames/s)	Standard deviation	Avg velocity (px/frame)	Max velocity (px/frame)	Most common emotion
Box	327	12.8	5.7	2.3	3.3	Neutral (intensity: low)
Donut	372	11.5	7.5	1.2	1.7	Disgust (intensity: high)
Pig	331	11.4	6.2	2.7	3.8	Happiness (intensity: moderate)
Spider	634	21.0	7.2	2.8	4.1	Fear (intensity: high)
Toy	447	15.5	5.8	1.6	2.3	Happiness (intensity: moderate)

Grasping frame rates are reported in frames/s; velocity columns summarize average and maximum frame-to-frame hand velocity (px/frame).

### 4.3 Grasping frame rate by object and modality (overview)

**Figure 5:**
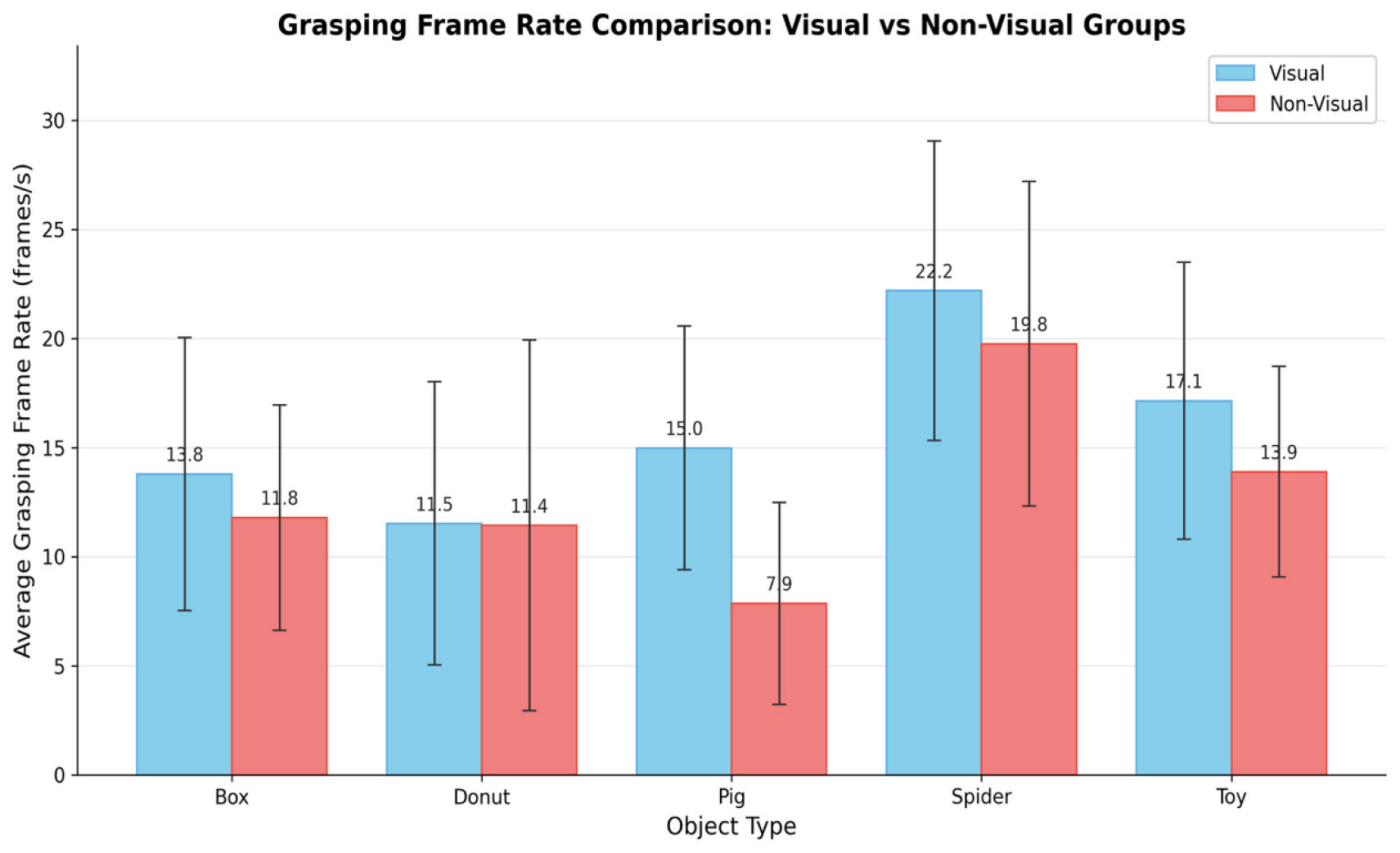
Grasping frame rate by object type for visual and non-visual groups. Error bars represent standard deviation across participants (*n* = 20 per group).

As an overview, Figure 5 shows the mean grasping frame rate for each object separately for the Visual and Non-Visual groups. The largest descriptive modality gap was observed for the pig (*M*_Visual_ = 14.99 vs. *M*_Non − Visual_ = 7.86 frames/s), suggesting that visual access substantially enhances grasping engagement for this object. In contrast, the spider maintained high grasping frame rates regardless of modality (*M*_Visual_ = 22.19, *M*_Non − Visual_ = 19.76), and the donut showed virtually no modality difference.

### 4.4 Overall group comparison (participant means)

**Figure 6:**
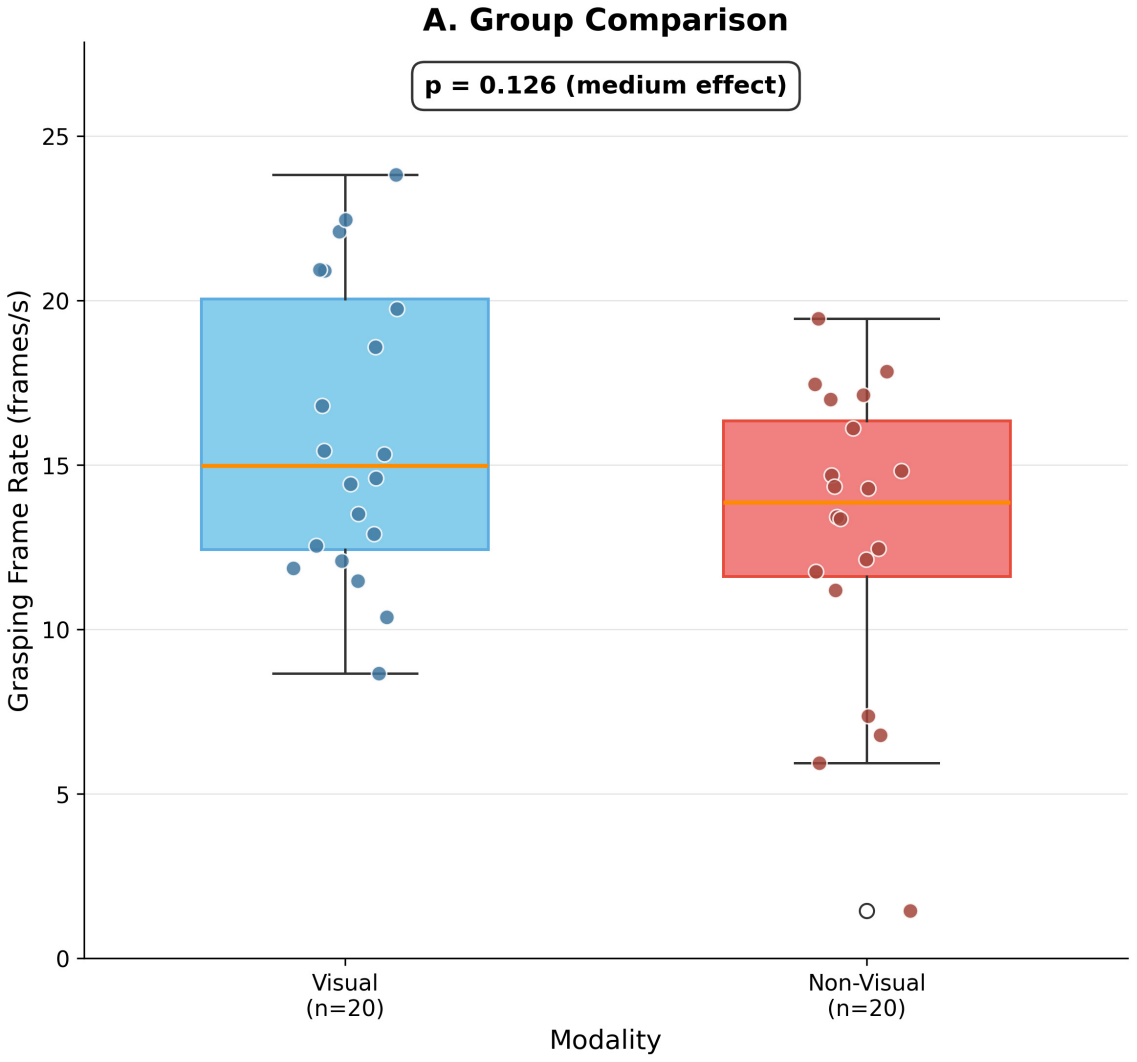
Overall group comparison using participant-level means. Each dot represents one participant; boxplots summarize the distribution (*n* = 20 per group).

At the participant level (mean across five objects), grasping frame rate was higher in the Visual group (*M* = 15.93 ± 4.48 frames/s) than in the Non-Visual group (*M* = 12.95 ± 4.57). This difference did not reach significance with a Mann–Whitney test (*U* = 257.0, *p* = 0.126; Figure 6), although the standardized effect size was medium (Cohen's *d* = 0.66; Cohen, 1988). The Mann–Whitney test served as a non-parametric robustness check (see Section 3.6). The mixed ANOVA (Table 2), reported alongside the LMM to allow comparison with prior literature, yielded a significant Modality main effect (*p* = 0.044). The two tests differ because the Mann–Whitney compares rank-ordered participant means, whereas the mixed ANOVA partitions within-subject variance across the five objects, which can yield greater power when its assumptions are met.

**Table 2:** Mixed ANOVA results for grasping frame rate (frames/s).

Source	SS	df	MS	*F*	*p*	η^2^p
Modality	442.96	1	442.96	4.33	0.044	0.102
Residual	3,887.40	38	102.30			
Object	2,577.10	4	644.27	26.61	< 0.001	0.412
Modality × object	269.08	4	67.27	2.78	0.029	0.068
Residual	3,679.92	152	24.21			

Modality is a between-subjects factor; object is a within-subjects factor. Effect sizes are reported as partial eta-squared (η^2^p).

### 4.5 Interaction effects on grasping frame rate

**Figure 7:**
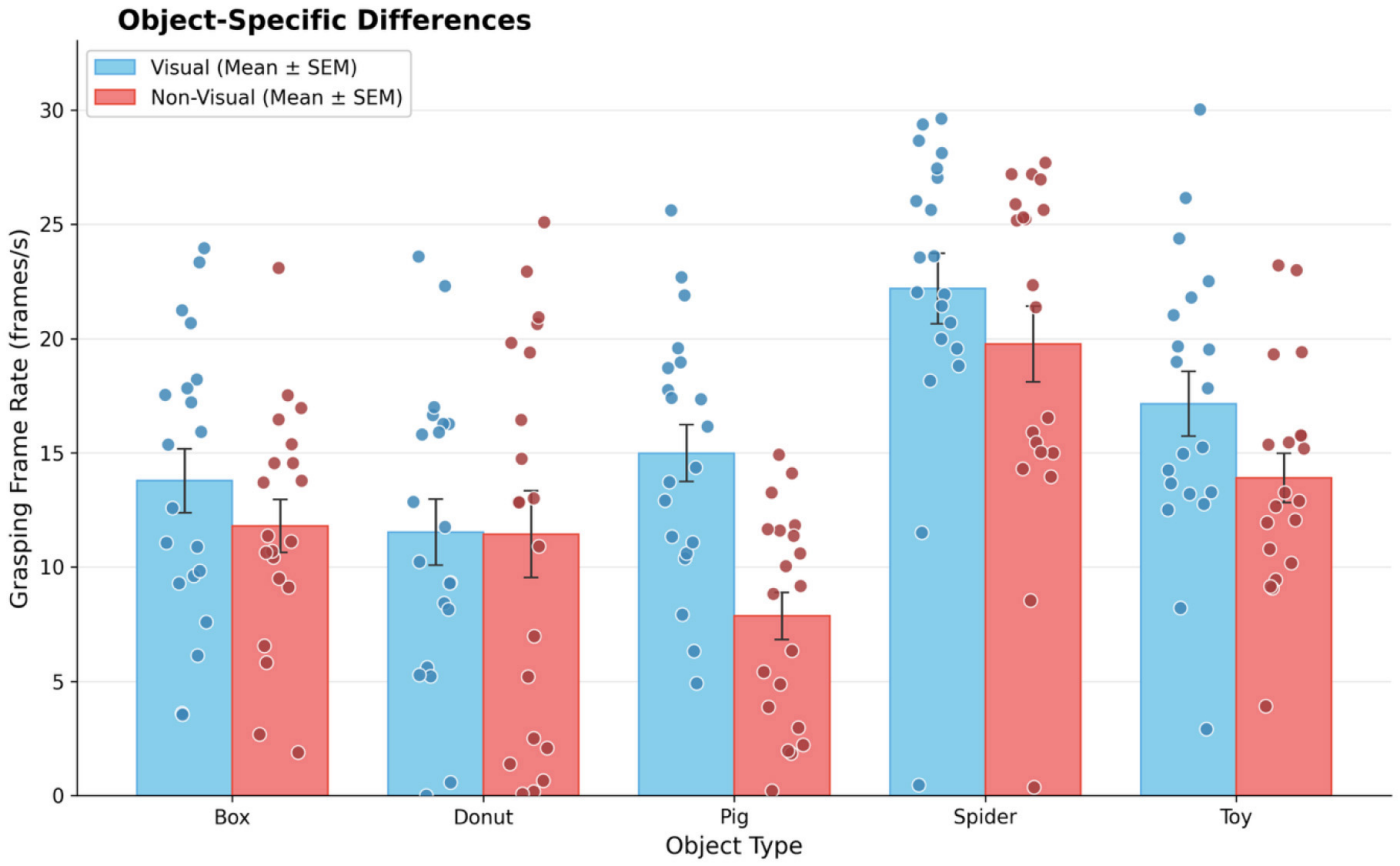
Object-specific differences between visual and non-visual groups. Bars show mean ± SEM; points show individual participant values (one observation per participant and object). Values are reported in frames/s.

Figure 7 shows grasping frame rate by object and modality (mean ± SEM with individual participant data points). The linear mixed-effects model revealed a significant Modality × Object interaction (likelihood-ratio test: χ^2^(4) = 11.291, *p* = 0.023), indicating that the effect of visual access depends on object type. The main effect of Object was highly significant, reflecting systematic differences in grasping frame rate across the five stimuli. The spider showed the strongest object effect (Spider vs. Box: +8.412 frames/s, *p* < 0.001). *Post-hoc* contrasts of modality within each object (Holm correction across five objects) showed a reliable difference only for the pig: Blind-Visual = −7.126 frames/s, 95% *CI* [−10.939, −3.313], *p*_Holm_ = 0.001 (Table 3). Object-wise Cohen's *d* values in the same Blind-Visual direction were: Box *d* = −0.35, Donut *d* = −0.01, Pig *d* = −1.39, Spider *d* = −0.34, and Toy *d* = −0.58. Table 2 presents the full mixed ANOVA results, including *F* values, degrees of freedom, and partial eta-squared effect sizes.

**Table 3:** *Post-hoc* modality contrasts within each object from the LMM (blind-visual).

Object	Visual mean	Blind mean	Blind-visual	95% CI	*p*_Holm_
Box	13.778	11.797	−1.981	[−5.795, 1.832]	0.634
Donut	11.530	11.439	−0.091	[−3.904, 3.722]	0.963
Pig	14.988	7.862	−7.126	[−10.939, −3.313]	0.001
Spider	22.190	19.759	−2.431	[−6.244, 1.382]	0.634
Toy	17.148	13.896	−3.253	[−7.066, 0.561]	0.378

Values are reported in frames/s; *p*-values are Holm-corrected across the five objects.

### 4.6 Movement category distribution

**Figure 8:**
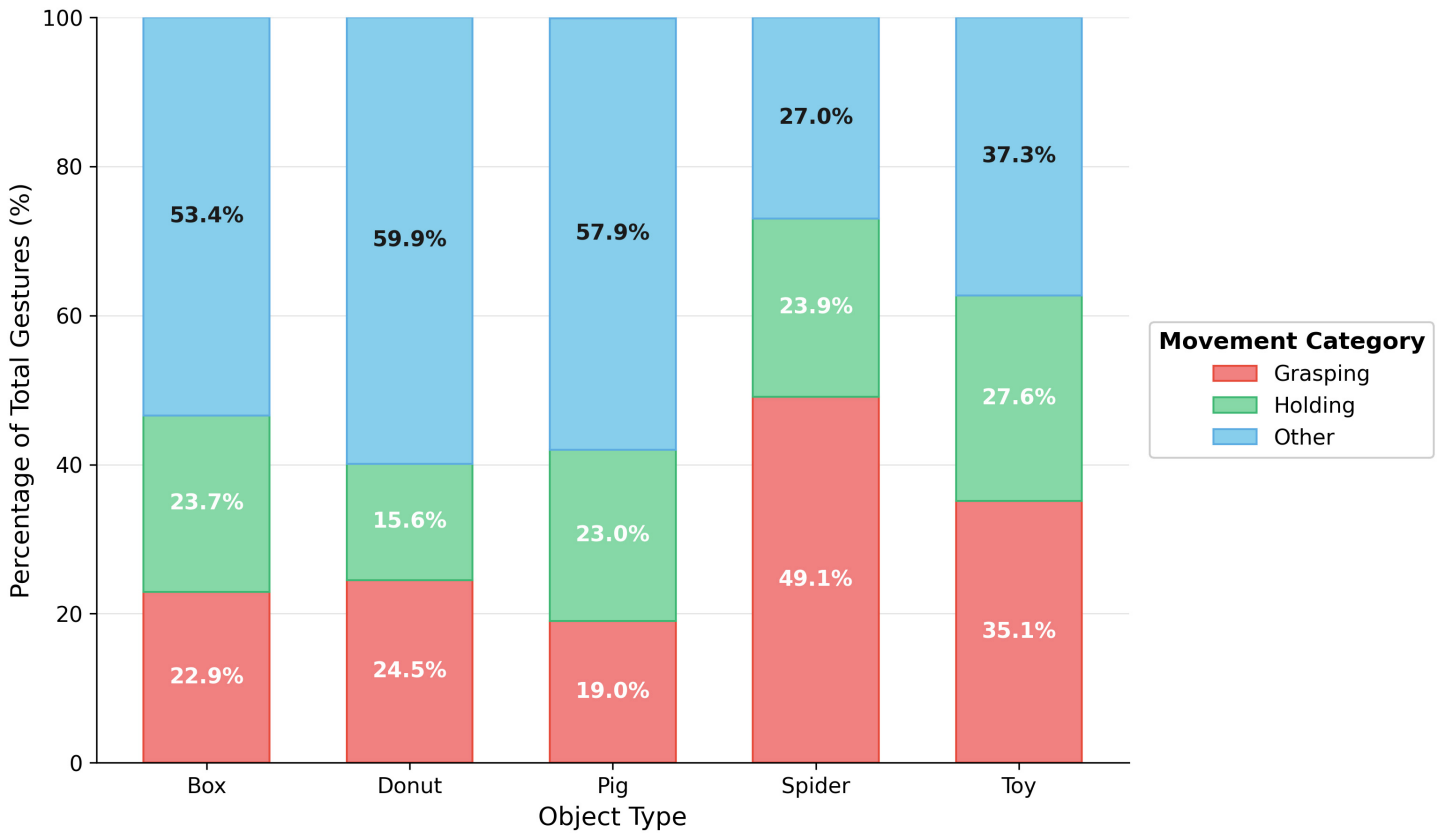
Distribution of movement categories by object type (all participants).

We grouped frame-wise labels into three movement categories: grasping (active manipulation), holding (sustained contact), and other movements (exploratory or transitional). Figure 8 shows the distribution across objects. The spider elicited the highest proportion of grasping frames (49.1%), whereas the box, donut, and pig were dominated by other movements (≥53%). These patterns complement the frame-rate results: the spider dedicated the largest share of interaction time to active grasping, while the donut and box profiles were dominated by non-grasping activity.

### 4.7 Emotion vs. grasping frame rate

**Figure 9:**
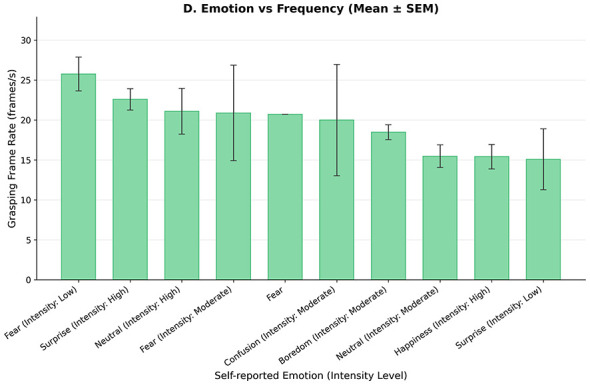
Grasping frame rate by self-reported emotion (top 10 emotion-intensity labels). Error bars indicate the standard error of the mean (SEM); values are reported in frames/s (s = second).

Figure 9 summarizes grasping frame rate across the ten most frequent emotion-intensity labels (mean ± SEM). Fear- and surprise-related labels tended to show higher grasping frame rate, whereas neutral and happiness labels were closer to the overall average. This pattern is consistent with the object-level findings and suggests that reported emotional intensity or alerting content, rather than valence direction alone, may partly relate to variation in grasping frame rate. Because category sizes were uneven (some labels occurred only a few times), this analysis is reported descriptively and should be interpreted with caution.

### 4.8 Velocity analysis

For hand velocity (px/frame), the same modeling strategy did not indicate a Modality × Object interaction (LRT χ^2^(4) = 0.918, *p* = 0.922), and the main effect of Modality was not significant (*p* = 0.355). This null finding indicates that emotional content selectively modulated grasping frame rate but not the spatial speed of hand movement. Overall, the dataset suggests that grasping frame rate was more sensitive to object and modality differences than velocity.

### 4.9 Bayesian robustness analyses

To complement the frequentist analyses and quantify the strength of evidence given the modest sample size, we conducted Bayesian Repeated Measures ANOVAs separately for grasping frame rate and hand velocity (see Methods, Section 3.6). Tables 4, 5 summaries the model comparison and inclusion Bayes Factors.

**Table 4:** Bayesian repeated measures ANOVA on grasping frame rate.

Model/effect	*P*(*M*|data)	BF_*M*_	BF_10_	BF_*inclusion*_
Null model	7.59 × 10^−15^	3.03 × 10^−14^	1.000	
Object	0.197	0.983	2.60 × 10^13^	
group	1.02 × 10^−14^	4.09 × 10^−14^	1.348	
Object + group	0.311	1.801	4.09 × 10^13^	
Object + group + object × group	0.492	3.879	6.49 × 10^13^	
Object (inclusion BF)	1.000			3.66 × 10^13^
Group/modality (inclusion BF)	0.803			2.714
Object × group (inclusion BF)	0.492			3.879

Default JZS priors (JASP 0.96). All models compared against the null model.

**Table 5:** Bayesian repeated measures ANOVA on hand velocity.

Model/effect	*P*(*M*|data)	BF_*M*_	BF_10_	BF_*inclusion*_	BF_01_
Null model	0.603	6.064	1.000		
Object	0.110	0.494	0.183		
Group	0.241	1.272	0.400		
Object + group	0.044	0.184	0.073		
Object + group + object × group	0.002	0.009	0.004		
Object (inclusion BF)	0.156			0.123	8.13
group/modality (inclusion BF)	0.287			0.269	3.73
Object × group (inclusion BF)	0.002			0.009	111.1

The null model is the best-fitting model. BF_01_ = 1/BF_*inclusion*_. Default JZS priors (JASP 0.96).

For grasping frame rate, the full interaction model (Object + Modality + Object × Modality) was the best-fitting model (*P*(*M*|data) = 0.492, BF_10_ = 6.49 × 10^13^) relative to the null model (Table 4). The inclusion Bayes Factors provided *decisive* evidence for the Object effect (BF_*inclusion*_ = 3.66 × 10^13^), *substantial* evidence for the Modality × Object interaction (BF_*inclusion*_ = 3.88), and *anecdotal-to-moderate* evidence for the Modality main effect (BF_*inclusion*_ = 2.71), the latter consistent with the borderline frequentist result for this term (*p* = 0.044).

For hand velocity, the null model was the best-fitting model (*P*(*M*|data) = 0.603; Table 5). The data therefore provided *decisive* evidence in favor of the null hypothesis for the Modality × Object interaction (BF_01_ = 111), and *substantial* evidence in favor of the null for the Object main effect (BF_01_ = 8.13) and the Modality main effect (BF_01_ = 3.73). Together, these analyses (Tables 4, 5) confirm that (i) the reported temporal-density effects on grasping frame rate are not artifacts of the modest sample size, and (ii) the velocity null result is a genuine null effect rather than a power-limited artifact, supporting the temporal–spatial dissociation interpretation outlined in Section 5.

## 5 Discussion

The primary objective of this study was to determine whether the emotional valence of an object is reflected in the kinematic profile of a natural grasp. By employing an accessible, vision-based tracking pipeline, we sought to capture subtle behavioral markers of affect that are often obscured in highly constrained laboratory settings. Our findings confirm that hand kinematics are not merely a product of biomechanical efficiency or task affordance, but are systematically modulated by the emotional properties of the target object. Crucially, the data suggest a dissociation between the spatial and temporal components of the reach. While the speed of the movement (velocity) remained relatively stable, the grasping frame rate shifted significantly in response to emotionally salient stimuli. This may indicate that emotionally salient objects alter the duration or density of grasp-related engagement, potentially reflecting heightened monitoring or cautious interaction rather than faster movement. This interpretation is consistent with a task-relevance account: in the present design, the emotional stimulus was also the object that participants had to grasp, making affective content directly relevant to the action goal (Montalti and Mirabella, 2023; Mirabella and Montalti, 2025).

### 5.1 Hypothesis validation: temporal vs. spatial modulation

To address our hypotheses and evaluate the specific mechanisms underlying this modulation, we reflect on our initial predictions in light of the unified dataset. Overall, the findings support the central premise that affective object content modulates natural grasping, but they refine the predicted mechanism. The first hypothesis was partially supported: affective object content modulated grasping frame rate, but not hand velocity. Thus, emotional modulation was expressed primarily in temporal density, reflecting sustained hand-object engagement, rather than in faster hand movement. The second hypothesis was also partially supported. Emotionally salient objects did not uniformly elicit more dynamic grasping behavior than neutral or comfort-associated objects. Instead, the effect was object-specific: the fear-inducing spider elicited the highest mean grasping frame rate (*M* = 20.97 frames/s), whereas the disgust-related donut did not show a comparable increase (*M* = 11.48 frames/s). Mixed-effects modeling confirmed a robust main effect for the spider relative to the neutral box object (Spider vs. Box: +8.41 frames/s, *p* < 0.001), indicating that threat-related object content was associated with especially dense grasp-related engagement. The effect also depended on sensory modality: the largest Visual–Non-Visual difference was observed for the pig, whereas the spider showed consistently high grasping frame rates in both modalities. These findings suggest that visual and non-visual access shape affective motor engagement in an object-specific manner, rather than through a single global arousal effect. They are consistent with theoretical accounts proposing that emotional valence shapes not only perceptual prioritization but also the frequency and structure of exploratory motor behavior (Lederman and Klatzky, 1987; Esteves et al., 2016). However, the emotion-by-frequency distribution (Figure 9) exhibited considerable variability and uneven sample sizes, warranting cautious interpretation of these patterns. Future work would benefit from restricting analyses to a smaller set of predefined emotional categories or incorporating physiological indices of arousal, such as skin conductance, to further disentangle subjective reports from affect-driven motor modulation.

### 5.2 Emotion-specific motor patterns

Rather than supporting a simple global arousal account, the distinct kinematic signatures for each object reveal how emotional content shapes motor behavior (Esteves et al., 2016). Fear-inducing stimuli (spider) elicited frequent and dense grasp-related contact, potentially reflecting defensive or exploratory responses to threat. Conversely, disgust-evoking objects (donut) produced minimal contact, consistent with avoidance behaviors (Rozin and Fallon, 1987). Neutral objects (box) showed intermediate patterns, whereas the pig and toy were most often associated with happiness and showed moderate-to-high grasping frame rate depending on the modality. These findings are therefore better interpreted as object- and affective-content-specific motor patterns, rather than as evidence for a single global mechanism. The opposing patterns for the pig (prolonged contact) and donut (reduced contact) are consistent with the proposal that cuteness and disgust operate as antagonistic forces on motor engagement (Sherman and Haidt, 2011). The spider's high activity may reflect evolutionary preparedness for threat detection (Öhman and Mineka, 2001; Flykt, 2006), while disgust-related avoidance serves protective functions against contamination (Curtis et al., 2011).

### 5.3 Visual vs. non-visual processing

The overall group comparison (participant means) did not reach significance, suggesting that tactile-driven interaction can elicit strong grasping behavior even without visual access. However, the significant Modality × Object interaction indicates that vision may still matter for specific objects. In particular, the pig showed a large and reliable visual advantage (Table 3), whereas the spider elicited high grasping frame rate in both modalities. Because the pig object had a visually cute and squeezable appearance, visual feedback in the Visual modality may have prolonged the interaction, consistent with evidence that viewing cute stimuli increases behavioral carefulness and fine-motor precision (Sherman et al., 2009; Nittono et al., 2012). In the Non-Visual modality, the pig was primarily experienced as a rubbery tactile object without visual appeal. This pattern suggests that visual-emotional appraisal may drive motor engagement differently than tactile-only appraisal. Therefore, we recommend that future research replicate the pig-specific effect and test whether it generalizes to other positive or “cute” stimuli. Additionally, incorporating neurophysiological measures, including EMG, EEG, and fMRI, during object interaction could help to elucidate the underlying neural mechanisms involved in emotion-driven motor responses.

The greater variability observed in the Non-Visual modality may reflect individual differences in tactile sensitivity or in emotional interpretation when visual cues are absent. Rather than being a limitation, this variability presents an opportunity to explore how individual differences in sensory processing styles contribute to emotion-motor coupling, potentially offering insight into personalized aspects of affective touch and motor control.

### 5.4 Methodological insights

Our smartphone-based motion tracking approach successfully captured emotionally relevant motor variations, demonstrating the viability of accessible technology for behavioral research. Our use of vision-derived landmarks aligns with trends identified in skeleton-based affect research, which reports robust affect cues in pose dynamics even without additional sensors (Lu et al., 2025). The key advantages of the proposed framework included: (1) the preservation of naturalistic hand movements within a controlled laboratory environment, and (2) the use of a non-intrusive motion capture system that enables accurate recording of hand kinematics without interfering with participants' behavior. The three-category movement classification (grasping, holding, other) effectively differentiated object-specific interaction patterns. The modal tendencies in participant responses broadly support our object selection strategy, while also showing that naturalistic objects can elicit mixed affective responses rather than perfectly uniform category labels. However, the predominance of neutral responses suggests that naturalistic settings may attenuate emotional responses compared to laboratory paradigms.

### 5.5 Limitations and future directions

Several limitations constrain interpretation. Although the unified dataset increased the sample size to *n* = 40, power may still be limited for small interaction effects and for analyses based on self-reported emotion categories with low counts. Because the stimuli were real objects rather than physically matched replicas, tactile properties such as rigidity, softness, texture, and elasticity may have contributed to responses, particularly in the Non-Visual modality. The interpretive scope of the temporal metric should also be treated cautiously: grasping frame rate indexes the amount of time classified as grasp-positive within the detected hand interval, but cannot by itself distinguish monitoring, freezing, exploration, or deliberate manipulation. Future studies should incorporate physiological measures (heart rate, skin conductance, and electromyography) to validate emotional arousal independent of self-report. Future replications should also consider dimensional affect instruments such as the Self-Assessment Manikin (SAM) to characterize valence and arousal more explicitly. The video analysis methodology, while accessible, lacks the precision of specialized motion capture systems. Direct force measurement would provide additional insights into grip strength variations. Longitudinal designs could examine whether emotion-motor coupling patterns remain stable across sessions or show habituation effects. Future work should explore individual differences in emotion-motor coupling, investigate developmental aspects of this relationship, and examine how cultural background influences emotional responses to objects. As a forward-looking direction, recent hand-object interaction work integrates vision-language models to predict future hand trajectories from natural-language task prompts; integrating such models (e.g., HandsOnVLM) could let us condition kinematic forecasts on verbalized goals and scene context, and test whether emotion-modulated grasps systematically deviate from language-specified plans (Bao et al., 2024).

## 6 Conclusion

This study demonstrates that emotional responses to objects systematically influence hand movement patterns during grasping tasks. Using accessible, smartphone-based motion tracking, we identified distinct kinematic signatures associated with objects evoking different emotions. In the unified dataset (*N* = 40), the fear-inducing spider elicited the highest grasping frame rate, whereas the pig and donut showed the lowest rates on average. Our findings contribute to the understanding of emotion-motor coupling in several directions. First, we provide evidence that object-specific emotional responses translate into measurable kinematic differences, supporting theories of embodied emotion. Second, we show that the influence of visual access is not purely global: while the overall Visual vs. Non-Visual difference was not significant, the mixed-model analysis revealed an object-specific interaction driven by a strong visual advantage for the pig. Third, velocity did not show a Modality × Object interaction, suggesting that grasping frame rate is a more sensitive readout than frame-based velocity in this paradigm. The practical implications extend to human-computer interaction, clinical assessment, and affective computing applications. Real-time kinematic analysis could enable emotion-aware technologies that respond to users' affective states through their natural hand movements. For clinical populations with limited verbal communication, kinematic assessment offers a non-invasive window into emotional processing. Future research should address the limitations identified here through larger samples, neuro-physiological and muscular validation, and examination of individual differences. By demonstrating that grasp-related hand dynamics can serve as an implicit readout of affect, this work provides a scalable foundation for future technologies that can perceive human emotion through the subtle cadence of physical interaction.

## Data Availability

The raw data supporting the conclusions of this article will be made available by the authors, without undue reservation.
